# Assessment of Risk Factors Associated With Coronary Artery Disease (CAD) in the Western Libyan Patients

**DOI:** 10.1155/cdr/1396974

**Published:** 2025-01-11

**Authors:** Osama Bheleel, Alaa Abdulhamid, Hajer Elmuaget, Hanaa Grash, Mohamed Hadi Mohamed Abdelhamid, Ibtisam Alhadi

**Affiliations:** ^1^Department of Cardiology, Tripoli University Hospital, Tripoli, Libya; ^2^Department of Statistics-Statistical Bioinformatics, Libyan Biotechnology Research Center (BTRC), Tripoli, Libya; ^3^Department of Research, Documentation and Scientific Committees, National Center for Disease Control, Tripoli, Libya; ^4^Department of Cell Biology and Tissue Culture, Libyan Biotechnology Research Center (BTRC), Tripoli, Libya

**Keywords:** coronary angiography, coronary artery disease, Libya, percutaneous coronary intervention, risk factors

## Abstract

Coronary artery disease (CAD) is the leading cause of death worldwide in both men and women. Accordingly, we retrospectively reviewed the effects of various risk factors on coronary angiographic outcomes. Data were collected from the catheter lab through Tripoli University Hospital records, whereas the team reviewed clinical data and coronary artery diagrams for 1 year from 01/04/2019 to 31/03/2020. In our study, the total number of cases was 666, 401 male and 265 female, aged between 27 and 91 years. Our analysis revealed a significantly higher incidence of CAD among male smokers under 60. Conversely, a majority of nonsmoker patients were female. The most common risk factors for women were diabetes mellitus (DM) and hypertension (HTN) (12% and 13%, respectively). While the men share the significant effects of smoking on coronary angiography (C. Angio) findings (40.52%), most of them underwent a percutaneous coronary intervention (PCI). In our study, there was evidence that CAD is a prevalent disease among middle-aged populations with male gender preference. The risk factors, including diabetes, HTN, and smoking, are the most contributing factors for developing CAD in Libya.

## 1. Introduction

Coronary artery disease (CAD) is the leading cause of death worldwide in both men and women. Approximately one in 30 patients with stable CAD experiences cardiovascular death or myocardial infarction (MI) each year [1]. It is the third leading cause of mortality worldwide and is associated with 17.8 million deaths annually. It also places a large economic burden on the community [1–3]. Moreover, little literature is related to the epidemiology of CAD in Africa. However, changes in lifestyle and modernization have raised the prevalence of CAD. Previous studies reported that the incidence of CAD is rising by 160% in the Middle East and North Africa, and the mortality rate from CAD was high (120 per 100,000 populations) [4, 5]. In Tunisia, where studies have reported CAD mortality rates increased by 11.8% for men and 23.8% for women between 1997 and 2009, further causing the death of 70% of cardiovascular patients [6, 7]. In addition, CAD has reached epidemic proportions in Egypt; the mortality rate due to CAD was measured to be 280 per 100,000 population [8].

There are several risk factors for CAD; some can be controlled, but not all. The nonmodifiable risk factors are age, sex, race, family history, and/or physiopathological conditions [9, 10].

Moreover, according to the 52-country case-control study, nine easily measurable, controllable, and/or modifiable risk factors account for more than 90% of the heart disease risk [11]. These factors include smoking, abnormal blood lipid levels, high blood pressure, diabetes, obesity, physical inactivity, poor diet, alcohol consumption, and psychosocial factors [11, 12]. Among these cases, CAD is more prevalent in men than in women. Additionally, other studies have highlighted that modifiable risk factors (excluding smoking) are more common in women and are associated with dietary patterns [13, 14].

There are three main strategies available for angina control and prevention or reversal of plaque progression: medical treatment, percutaneous coronary intervention (PCI), and/or coronary artery bypass grafting (CABG) [15].

PCI is often part of standard therapy in patients presenting with significant CAD. Similarly, in patients with stable CAD, PCI can be considered a valuable initial mode of revascularisation in all patients with objective extensive ischaemia in the presence of almost every lesion subset, with only one exception: chronic total occlusions that cannot be crossed [16].

Since 1977, Grundzig performed the first PTCA in Zurich, and PCI has been recognized as a leading procedure in treating CAD patients and has become a common part of routine practice worldwide [17].

Correspondingly, CABG is an acceptable procedure used to treat CAD and manage refractory to medical treatment. Globally, there are around one million patients who undergo CABG surgery each year [18].

In Libya, the coronary angiography (C. Angio) procedure has rapid expansion and uptake. However, when considering the entire clinical practice in Libya, limited data are available to describe nationwide contemporary practice patterns of C. Angio. Yet, until now, no unified nationwide registry has been established to demonstrate the accurate figures of cardiac procedures. Despite this, there has been a limited routine collection of related data, particularly around quality, safety, and cost. Concerning the growing need for C. Angio, the development of C. Angio registries in Libya was of growing interest. As a clinical quality registry, a C. Angio database is an essential mechanism for monitoring and benchmarking the performance of clinical care, improving safety and outcomes, contributing to reducing treatment costs and regulating guidelines.

In this context, this study was aimed at describing the characteristics and outcomes of consecutive unselected Libyan patients who underwent diagnostic C. Angio in a nationwide cohort of 1 year. The present study is the first part of a study of CAD risk factors and causes in Libyan patients. Also, a preprint has been previously published [19].

## 2. Material and Method

### 2.1. Study Population

The present study is the first part of a study of CAD risk factors and causes in Libyan patients. The total number of patient records reviewed who underwent diagnostic C. Angio during this study was 666 cases. However, 51 cases were dropped due to personal reasons; hence, the data on these cases were excluded. Therefore, 615 cases were contributed to this study: 371 male and 244 female. The age range of the study population was 27–91 years. C. Angio patients' reports were revised in the catheter lab (cath lab) of Tripoli University Hospital (TUH).

### 2.2. Data Collection

The data was collected from the cath lab from TUH records; the team reviewed and collected all clinical data of patients that were measured at admitted days, such as BMI, family history, lipid profiles, age, gender, and chronic diseases; we also reviewed all coronary artery diagrams during 1 year from 01/04/2019 to 31/03/2020 retrospectively. Additionally, essential clinical data related to physical activity, dietary behavior, and physiopathological conditions is unavailable.

#### 2.2.1. Data Availability

The data collection for our study was constrained by the operational challenges posed by the ongoing conflict in the region. Being the only functional medical facility during this period, the hospital had limited resources and faced significant logistical issues that impacted our ability to collect a full spectrum of data.

#### 2.2.2. Focus on Available Data

Despite these challenges, we prioritized the collection of data that was readily available and most critical for the immediate diagnosis and management of patients presenting to the cath lab. This included age, sex, hypertension (HTN), diabetes mellitus (DM), and smoking status, which are well-established risk factors for CAD and were feasible to document under the circumstances.

### 2.3. Ethics Statement

The Libyan National Committee for Biosafety and Bioethics (LNCBB) approved the study protocol (N: 37/SH/21). All participants provided informed consent, and the study adhered to the principles outlined in the Declaration of Helsinki [20].

### 2.4. Statistical Analysis

Continuous variables were presented as mean ± SD and compared using two-way variance analysis. We categorized participants based on their age into three groups: (1) ≤ 40 years, (2) 40–60 years, and (3) ≥ 60 years. Additionally, we classified risk factors into two categories: (1) modifiable risk factors (e.g., high blood pressure, smoking, and diabetes) and (2) nonmodifiable risk factors (e.g., gender and advancing age). In all cases, *p* values < 0.05 were considered statistically significant. All data were processed using the Statistical Package for Social Sciences, Version 25 (SPSS, Chicago, IL, United States).

Moreover, we have included a statement in the manuscript indicating that the Strengthening the Reporting of Observational Studies in Epidemiology (STROBE) checklist was used to guide the reporting of this study.

## 3. Results

The total number of cases is 615: 371 (60.32%) men and 244 (39.67%) women; the mean age of the study population was 59.4 ± 13.4 years (an age range of 27–91 years).

We recorded no classical risk factors with 276 patients, representing 44% of total cases. Furthermore, these cases were dilated cardiomyopathy, left bundle branch block, and preoperative assessment for cardiac surgery.

### 3.1. Nonmodifiable Risk Factors for CAD

Our findings revealed a significantly higher percentage of men (55.19 ± 10.2; *p* ≤ 0.05) underwent C. Angio compared to women. The majority of participants were aged 40–60 (52.92%), with a significantly higher proportion of men in this age group undergoing C. Angio (56.42% vs. 48.55%; *p* ≤ 0.05). The demographic characteristics and clinical data of the participants are summarized in Table 1.

### 3.2. Modifiable Risk Factors for CAD

According to the results of this study, the most common risk factors for women were DM, HTN, and women who have DM+HTN (12.8%, 13.9%, and 52.17%, respectively). However, no cases of smoking have been reported among women (Table 1).

The prevalence of men's risk factors was as follows: smoking (36.7%), HTN (14.11%), and DM (12.04%), which were lower compared to women's cases at the last two factors (Table 1). The incidence of smoking was significantly higher than the other risk factors.

### 3.3. Result of C. Angio

**Figure 1:**
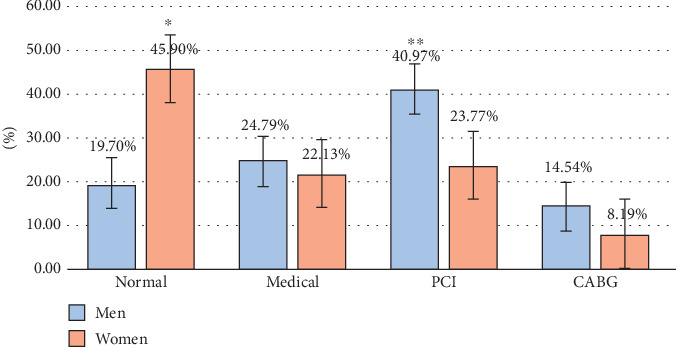
Effect of gender on result of C. Angio. PCI: percutaneous coronary intervention; CABG: coronary artery bypass grafting. ⁣^∗^High significant difference (a *p* value < 0.05 was considered significant). ⁣^∗∗^Very high significant difference (a *p* value < 0.05 was considered significant).

**Figure 2:**
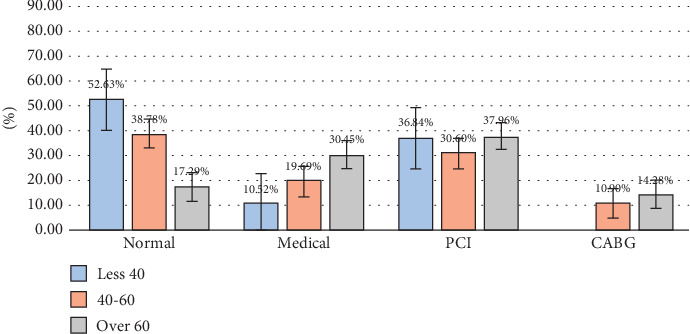
Effect of the age factor on the result of C. Angio. PCI: percutaneous coronary intervention; CABG: coronary artery bypass grafting.

The results of C. Angio showed that out of 186 cases (30%), the majority did not require medical or surgical intervention, and these cases were predominantly women. Simultaneously, the data showed that the percentage of men who underwent medical, PCI, and CABG were 24.74%, 40.97%, and 14.55%, respectively. The incidence of PCI cases was one and a half times higher for men than for women. The PCI and CABG procedures increased with age and included ages > 40 years in men and > 60 years in women (Figures 1 and 2).

**Figure 3:**
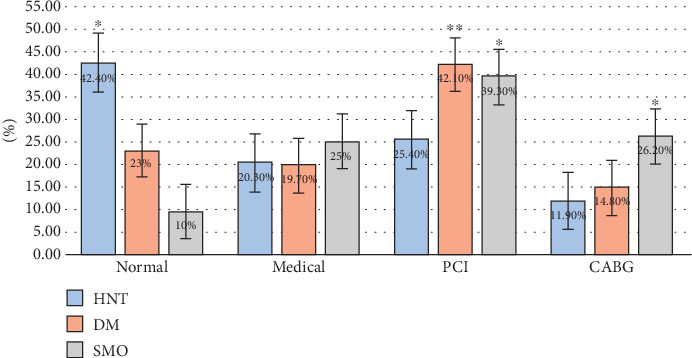
C. Angio results with different risk factors. PCI: percutaneous coronary intervention; CABG: coronary artery bypass grafting. ⁣^∗^High significant difference (a *p* value < 0.05 was considered significant). ⁣^∗∗^Very high significant difference (a *p* value < 0.05 was considered significant).

Furthermore, our data show that risk factors significantly influenced PCI more than others. Approximately 15% of all CABG was performed on men with diabetes, while strong evidence was found that men who underwent a PCI were smokers (42%) (a *p* value < 0.05 was considered significant) (Figure 3).

## 4. Discussion

The current study represents the first Libyan CAD enrollment and the result of C. Angio for patients managed in the public sector in university and regional hospitals. Over half of the patients who underwent C. Angio were men (60.32%), which is to be expected because CAD is a primarily men's gender disease. This is consistent with the data obtained by Adda et al. [6], who found that patients who underwent C. Angio are more men than women (88.5% vs. 21.5%, respectively) [6].

The second strong observation is that most cases that underwent C. Angio fall into the age group between 40 and 60 years, and after that, the patients are over 60 years old. Additionally, the hypothesis of different previous studies showed that in women under 55, the incidence of CAD is one-third that of men. However, it gradually increases with age when crossing over 55 years. This finding is widely supported by existing research [21–23].

Physiological differences between the two genders suggest that age-related changes in endogenous sex hormones, such as estrogen, progesterone, and androgens, play a pivotal role in controlling CAD. Exceptionally, the hormone estrogen plays an essential role as a cofactor in the onset of CAD in women [24, 25].

CAD is the disease of the middle age group, which is the product group in the community. This fact has an impact on the productivity and performance of the age group mentioned earlier; as a matter of fact, this will be reflected in the community in various aspects, such as absences from work, the cost of health care services, expensive medical and device therapy, and the future outcome in the form of chronic heart failure and its sequel. Those facts highlight the prevention strategies and education of the community starting at early age groups, especially among adolescents aiming to reduce the burden of CAD, which is the cornerstone in any health strategies for the Ministry of Health.

Moreover, CAD affects all groups of the study as a result of modernization associated with the modifiable risk factors for CVD, including diabetes, high blood pressure, smoking, and both risk factors.

In addition, more than half of our population sample had at least one significant risk factor. Regarding the risk factors for CAD, about one-third of the cases were diabetics and hypertensive or were having both. As both DM and HTN are prevalent diseases in our community, tighter glycemic control of people with diabetes and targeting high blood pressure will result in a reduction in the incidence and prevalence of CAD in the community [26–28].

So far, in men, smoking is one of the major risk factors for cardiovascular disease causes and is a leading cause of death. This effect is very prevalent. It should be noted that the percentage of Libyan smokers aged between 25 and 64 years among men was very high at 49.6% [29]. We believe that the high level of Libyan smokers increased the number of men who underwent C. Angio in our study.

It also appears that exposure to multiple factors simultaneously increases the required medical or PCI intervention if compared to typical C. Angio results.

Considering the results of the C. Angio in our studied population, over 40% of the studies revealed normal C. Angio, and most of them were women (woman patients who underwent the C. Angio have normal epicardial coronary arteries); this again highlights that screening for CAD among the female gender ought to be re-evaluated to use more noninvasive modalities of screening for CAD, such as stress echocardiography or CT-C. Angio, and not to depend only on exercise ECG for sending patients for C. Angio, as it has high false-positive results. 34.1% of the cases underwent PCI implantation; the most stented single artery is the left anterior descending artery at 12.8% (data not shown).

### 4.1. Limitations of Study

Our retrospective study encountered significant challenges due to poor conditions during data acquisition. Notably, the absence of an accurate archiving system for coronary angiograms and inadequate recording of personal patient data—such as missing contact telephone numbers—compounded these difficulties. Moreover, essential data on cardiac risk factors were often unrecorded, and procedural details like the approach (femoral or radial), medications administered during the procedure, and any immediate or remote complications were not systematically registered. Crucially, the ongoing conflict in Libya exacerbated these issues, severely hindering our ability to collect comprehensive data. The conflict resulted in operational interruptions and resource constraints, which impeded our research activities. This included power and internet outages, working under emergency conditions, and transportation restrictions. As a result, many patients' data could not be collected or were incomplete. Yet, without electronic archiving, our study relied solely on paper records, which were not maintained professionally. This reliance on an unorganized paper archiving system further negatively impacted the quality and completeness of our data.

## 5. Conclusion

In our study, we found compelling evidence that CAD is prevalent among middle-aged populations, with a male gender preference. Clinical data indicated that the Libyan adult population has a high level of CAD risk factors, including diabetes, HTN, and smoking, which are the most contributing factors to the development of CAD; those risk factors are modifiable and preventable direct causes of CAD, which may require urgent decision-making to address national control measures regarding CAD.

Establishing a national registry for coronary procedures is crucial and mandatory for ensuring patient safety and maintaining quality control in cath labs.

## Figures and Tables

**Table 1:** Prevalence of risk factors stratified by modifiable and nonmodifiable risk factors.

	**%** **p** **a** **t** **i** **e** **n** **t** **s** ± **SD (N** = 308**)**	**%** **m** **e** **n** ± **SD (N** = 170**)**	**%** **w** **o** **m** **e** **n** ± **SD (N** = 138**)**
Age (years)			
≤ 40	2.92 ± 0.10	3.52 ± 0.04	2.17 ± 0.10
40–60	52.92 ± 0.19	56.42 ± 0.30^∗^	48.55 ± 0.23
≥ 60	44.15 ± 0.26	40 ± 0.23	49.27 ± 0.31^∗∗^
Risk factor			
HTN	3.15 ± 0.86	14.11 ± 0.54	18.83 ± 0.09^∗^
DM	7.80 ± 0.90	12.04 ± 0.24	29.03 ± 0.11^∗^
Smoke	40.52 ± 0.97^∗∗^	36.47 ± 0.47^∗∗^	0
HTN+DM	21.93 ± 0.75^∗^	18.65 ± 0.17	52.14 ± 0.36^∗∗^
HTN+smoke	11.99 ± 0.12	9.41 ± 0.11	0
DM+smoke	8.84 ± 0.61	5.88 ± 0.34	0
HTN+DM+smoke	5.97 ± 0.34	3.52 ± 0.04	0

*Note: N*: number of patients who have risk factors.

Abbreviations: DM: diabetes mellitus; HTN: hypertension.

⁣^∗^High significant difference (a *p* value < 0.05 was considered significant).

⁣^∗∗^Very high significant difference (a *p* value < 0.05 was considered significant).

## Data Availability

Data is available in the manuscript.
